# Acute cellular rejection in lung transplantation

**DOI:** 10.7196/AJTCCM.2019.v25i2.010

**Published:** 2019-07-31

**Authors:** F Manyeruke, T Pennel, R Roberts, G L Calligaro

**Affiliations:** 1 Division of Pulmonology, University of Cape Town Lung Institute and Groote Schuur Hospital, Cape Town, South Africa; 2 Chris Barnard Division of Cardiothoracic Surgery, University of Cape Town and Groote Schuur Hospital, Cape Town, South Africa; 3 Division of Anatomical Pathology, National Health Laboratory Service and University of Cape Town, South Africa

**Keywords:** cellular rejection, lung transplantation

## Abstract

Lung transplantation is an important therapy for end-stage respiratory failure in patients who have exhausted other therapeutic options. The lung is unique among solid-organ transplants in that it is exposed to the outside environment, and undergoes continuous stimulation from infectious and non-infectious agents, which may play a part in upregulating the immune response to the allograft. Despite induction immunosuppression and the use of aggressive maintenance regimens, acute allograft rejection is still a major problem, especially in the first year after transplant, with important diagnostic and therapeutic challenges. As well as being responsible for early graft failure and death, acute rejection also initiates alloimmune responses that predispose patients to chronic lung allograft dysfunction, in particular bronchiolitis obliterans syndrome. Cellular responses to human leukocyte antigens (HLAs) on the allograft have traditionally been considered the main mechanism of acute rejection, although the influence of humoral immunity is increasingly recognised. Here, we present two cases of acute cellular rejection (ACR) in the early post-transplant period and review the pathophysiology, diagnosis, clinical presentation and treatment of ACR.

## Background

Lung transplantation is rapidly gaining traction as a viable procedure for patients with end-stage lung disease and limited life expectancy. Its availability has spread to an expanding number of countries worldwide, including, more recently, to the state sector in South Africa. Despite advances in immunosuppression, acute rejection of the pulmonary allograft remains a major problem. The lung has the highest rate of rejection among transplanted solid organs, and as much as half of all lung transplant recipients will be treated for allograft rejection in the first year after transplant.^[1]^ Acute cellular rejection (ACR) is the most common form of allograft rejection and is a serious complication not only because it can lead to acute graft dysfunction or failure, but also because it is a major risk factor for the development of chronic lung allograft dysfunction,^[2]^ particularly the bronchiolitis obliterans syndrome (BOS).

## Cases

Here we describe two patients with bilateral lung transplants with acute cellular rejection in the early post-transplant course. Both patients were female, aged 18 and 58 years, transplanted for cystic fibrosis and chronic obstructive pulmonary disease, who presented acutely with cough and shortness of breath at 11 and 9 weeks post transplant, respectively.

Both patients had negative crossmatches at the time of transplant, and received induction immunosuppression with an interleukin-2 receptor inhibitor (basiliximab). Maintenance immunosuppression consisted of a calcineurin inhibitor (tacrolimus), cell cycle inhibitor (in the first patient, mycophenolate mofetil, and in the second, azathioprine), and prednisolone at tapering doses. Previous protocol-driven surveillance transbronchial biopsies at 3-week intervals had not shown any evidence of rejection.

**Fig. 1:**
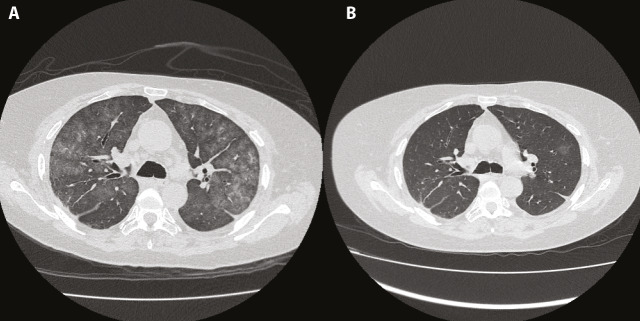
High-resolution computed tomography scan of the chest. (A) Pre-treatment scan showing extensive, bilateral ground-glass opacities (GGO) and (B) in the resolution phase, after treatment with corticosteroids, showing improvement in the GGO globally but with persistence of some centrilobular ground-glass nodules in the right lung posteriorly.

Radiological changes at presentation included extensive bilateral ground-glass infiltrates affecting the mid- to lower lobes, with mild interlobular septal thickening, and some centrilobular nodularity (Fig. 1). Spirometry showed a deterioration in forced expiratory volume in 1 second (FEV_1_) of more than 10% from baseline. Peripheral blood differential counts performed on both patients did not show an elevation in eosinophil count, lymphocyte count or basophil count.

Transbronchial lung biopsy was diagnostic of acute cellular rejection in both patients. The Lung Rejection Study Group (LRSG) of the International Society for Heart and Lung Transplantation has standardised the diagnostic criteria and grading for ACR (Table 1).^[3]^

**TABLE 1:** Pathological grading of acute cellular rejection*

	**Grade**	**Meaning**	**Appearance**
**A-grade: Perivascular** **inflammation**	0	None	Normal lung parenchyma.
	1	Minimal	Scattered, infrequent small mononuclear perivascular infiltrates.
			No eosinophils.
	2	Mild	More frequent perivascular infiltrates identifiable at low magnification.
			Eosinophils may be present.
	3	Moderate	Dense perivascular infiltrates, eosinophils and neutrophils common.
			Pathognomonic feature is extension into alveolar septae and airspaces.
	4	Severe	Diffuse perivascular, interstitial and air-space infiltrates with pneumocyte damage and features of acute lung injury.
**B-grade: Airway-associated** **inflammation**	0	None	No evidence of bronchiolar inflammation.
	1R	Low grade	Single-layer mononuclear cells in bronchiolar submucosa.
	2R	High grade	Larger infiltrates of larger and activated lymphocytes in bronchiolar submucosa, with potential involvement of eosinophils and plasmacytoid cells.
	X	Ungradable	No bronchiolar tissue available.

R = revised

*Adapted from the 2007 Working Formulation of the International Society for Heart and Lung Transplantation.^[3]^

**Fig. 2:**
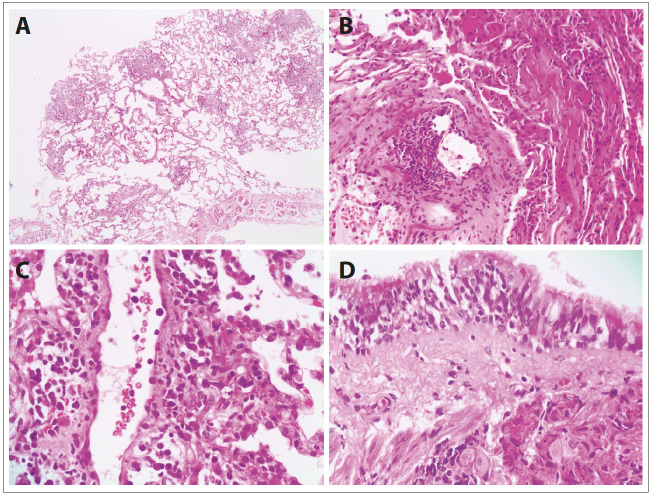
Transbronchial lung biopsies (haematoxylin and eosin staining). (A) Well-expanded airspaces with discrete perivascular cellular infiltrates, grade A2 (40×). (B) Multiple layers of lymphocytes surrounding a pulmonary venule, grade A2 (200×). (C) Sub-endothelial lymphocytes expanding an alveolar septum and associated endothelialitis grade A3. (D) A bronchiole with scanty submucosal lymphocytes showing grade B1R rejection (400×).

Perivascular inflammation, termed A-grade, evaluates the presence and extent of mononuclear cell infiltration around the blood vessels, surrounding submucosal interstitium and alveolar walls. The grades range from A0 (no rejection) to A4 (severe). Airway inflammation, termed B-grade rejection, evaluates the lymphocytic response in the submucosa of bronchioles, which may extend through the basement membrane at higher grades. In the first patient, the histological grade was A2B0, and in the second, the histological grade was A3B1R (Fig. 2). Both A and B grades range from 0 (no rejection) to 4 (severe) and are independent of each other.

Infection was rigorously ruled out by culture and nucleic acid testing of bronchoalveolar lavage fluid for bacteria, *M. tuberculosis* and fungi. A screen for circulating IgG donor-specific HLA antibodies (DSA) was negative in both patients.

The rejection episodes were treated with a tapered course of oral prednisolone over 4 weeks; the second patient also received a preceding 3-day pulse of methylprednisolone due to the high grade of rejection. Both patients showed radiological resolution (Fig. 1B) and an improvement in FEV_1_ to baseline post treatment.

Follow-up bronchoscopy performed after 6 weeks of treatment showed histological resolution of ACR.

## Discussion

The frequency and severity of ACR are the most significant risk factors for the development of BOS. BOS is, directly and indirectly, responsible for most lung transplant deaths after 1 year. The median survival among patients with lung transplant is 5.8 years, and 5- and 10-year survivals are 54% and 32%, respectively.^[4]^

ACR occurs mainly in the first 6 months after lung transplantation and is T-lymphocyte driven. Allorecognition occurs via the direct and the indirect pathway. The direct pathway involves the donor dendritic cells presenting an intact major histocompatibility complex (MHC) antigen to the recipient T cells. In the indirect pathway, recipient dendritic cells process and present alloantigen from the graft directly to recipient T cells.^[5]^

Currently, there are no ACR-specific laborator y findings. Elevations in eosinophils, basophils and lymphocytes have been proposed as possible markers of rejection while elevated neutrophil count points towards infection;^[6]^ however, we did not observe this in our patients.

Spirometry monitoring in lung transplant recipients is a sensitive way of monitoring patients for the presence of rejection or infection. Spirometry can be performed on routine hospital visits or at home with telemetry monitoring. A decrease in FEV_1_ by 10% has been shown to have a sensitivity of 60 - 75% in the diagnosis of ACR but this has low specificity, mainly because infections are also associated with a decrease in FEV_1_ .^[7,8]^

Surveillance bronchoscopies are performed at many centres (including our own) and have been shown to diagnose subclinical ACR in up to 25% of biopsies. This is usually of low grade (A1B0); the incidence of grade A2 or higher rejection is only 16%, and B1R or higher is 14%.^[7,9]^ However, the practice of surveillance biopsy is controversial because early detection of low-grade rejection does not seem to alter outcome,^[1,10]^ transbronchial biopsy is not without risk, A1B0 rejection may resolve spontaneously, and effect of treatment of these episodes on long-term outcomes is unclear. In our two cases, ACR was diagnosed with clinically indicated bronchoscopies, and all surveillance bronchoscopies were normal.

The treatment of ACR depends on institutional practice and the histological grade of rejection. The cornerstone of treatment for ACR is the steroid pulse; however, there is a paucity of evidence to guide the dose or duration of therapy. Most cases of high-grade rejection (A2 and above) will be treated with pulseddose methylprednisolone for ~3 days with the transition to a tapering oral steroid wean. Some clinicians will use only oral prednisolone (0.5 - 1.0 mg/kg) for milder grades of rejection, although evidence for this practice is also lacking. In our centre, oral prednisone at 1 mg/kg is used for most cases of A1 and all cases of A2 rejection, and pulsed methylprednisolone for more severe grades. Repeat bronchoscopy is performed at 6 weeks to assess for resolution of ACR.

The management of persistent or refractory ACR is not well researched. Failure to respond to treatment for ACR should trigger investigations for concomitant antibody-mediated rejection (AMR). Together with a repeat steroid pulse, other changes to therapy in cases of persistent ACR may include a class-switch of calcineurin inhibitor (usually from cyclosporine to tacrolimus),^[11,12]^ or the addition of a mammalian target of rapamycin (mTOR) inhibitor, such as everolimus. Other therapies include alemtuzumab (an antibody to CD52) and extracorporeal photopheresis. Locally most patients are on tacrolimus and everolimus is the preferred add on treatment.

This report has focused on acute rejection as a cellular driven immune response, which has historically been considered the main mechanism of acute lung allograft rejection. However, over the past decade, the role played by antibodies generated against the allograft has stimulated growing interest, and AMR has evolved from a hypothetical and controversial concept to a crucial diagnostic consideration in patients with acute allograft dysfunction, and a well-recognised clinical entity post lung transplantation.^[13–15]^

**Fig. 3:**
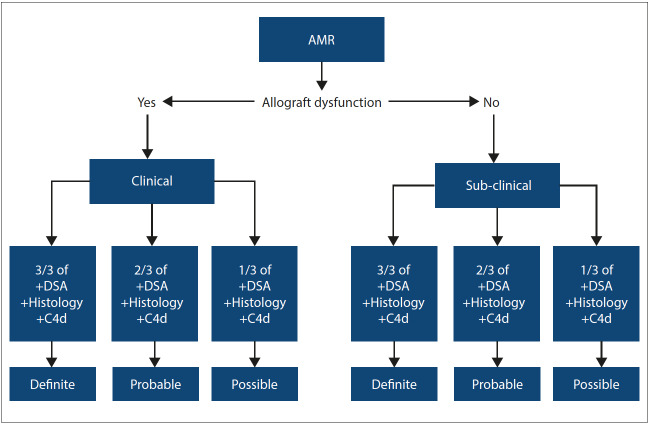
Classification of AMR.^[14]^ AMR = antibody-mediated rejection DSA = donor-specfic antibodies C4d = complement 4d

AMR occurs when allospecific B cells and plasma cells produce donor-specific antibodies (DSAs). DSAs form antigen-antibody complexes which cause lung tissue pathology and graft dysfunction via both compliment-dependent and independent mechanisms. The diagnosis of AMR requires clinical vigilance and requires a multi-modal approach, with very few cases, meeting the full criteria, which include: graft dysfunction, positive DSA with histopathological features of capillaritis with complement 4d (C4d) staining on histology, and exclusion of an alternative diagnosis. A diagnosis of AMR can be clinical or sub-clinical based upon presence or absence of allograft dysfunction; it is also classified as definite, probable and possible (Fig. 3). Currently, there is no definite treatment protocol for AMR in lung transplant, and treatment includes plasmapheresis, intravenous human immunoglobulin, rituximab and bortezomib.^[14]^

## Conclusion

ACR remains a significant challenge among patients who have undergone lung transplantation. Vigilant clinical monitoring of patients and performing clinically indicated bronchoscopy remain the best means for early diagnosis. There is a great need for a non-invasive biomarker to assist in predicting patients at risk of developing ACR. Current immunosuppressive therapies targeting T-cell responses do not universally prevent ACR or the development of chronic lung allograft dysfunction.

## References

[1] Martinu T, Chen DF, Palmer SM (2009). Acute rejection and humoral sensitization in lung transplant recipients.. Proc Am Thorac Soc.

[2] Benzimra M, Calligaro GL, Glanville AR (2017). Acute rejection.. J Thoracic Dis.

[3] Stewart S, Fishbein MC, Snell GI (2007). Revision of the 1996 working formulation for the standardization of nomenclature in the diagnosis of lung rejection.. J Heart Lung Transplant.

[4] Yusen RD, Edwards LB, Kucheryavaya AY (2015). The Registry of the International Society for Heart and Lung Transplantation: Thirty-second Official Adult Lung and Heart-Lung Transplantation Report – 2015. Focus Theme: Early Graft Failure.. J Heart Lung Transplant.

[5] Hsiao HM, Scozzi D, Gauthier JM, Kreisel D (2017). Mechanisms of graft rejection after lung transplantation.. Curr Opin Organ Transplant.

[6] Speck NE, Schuurmans MM, Murer C, Benden C, Huber LC (2016). Diagnostic value of plasma and bronchoalveolar lavage samples in acute lung allograft rejection: Differential cytology.. Respir Res.

[7] McWilliams TJ, Williams TJ, Whitford HM, Snell GI (2008). Surveillance bronchoscopy in lung transplant recipients: Risk versus benefit.. J Heart Lung Transplant.

[8] Otulana BA, Higenbottam T, Ferrari L, Scott J, Igboaka G, Wallwork J (1990). The use of home spirometry in detecting acute lung rejection and infection following heart-lung transplantation.. Chest.

[9] Glanville AR, Aboyoun CL, Havryk A, Plit M, Rainer S, Malouf MA (2008). Severity of lymphocytic bronchiolitis predicts long-term outcome after lung transplantation.. Am J Respir Crit Care Med.

[10] Benzimra M (2018). Surveillance bronchoscopy: Is it still relevant?. Semin Resp Crit Care Med.

[11] Sarahrudi K, Estenne M, Corris P (2004). International experience with conversion from cyclosporine to tacrolimus for acute and chronic lung allograft rejection.. J Thoracic Cardiovasc Surg.

[12] Vitulo P, Oggionni T, Cascina A (2002). Efficacy of tacrolimus rescue therapy in refractory acute rejection after lung transplantation.. J Heart Lung Transplant.

[13] Hachem RR (2017). Acute rejection and antibody-mediated rejection in lung transplantation.. Clin Chest Med.

[14] Levine DJ, Glanville AR, Aboyoun C (2016). Antibody-mediated rejection of the lung: A consensus report of the International Society for Heart and Lung Transplantation.. J Heart Lung Transplant.

[15] Westall GP, Snell GI (2014). Antibody-mediated rejection in lung transplantation: Fable, spin, or fact?. Transplantation.

